# Correlation of intratumoral mast cell quantity with psychosocial distress in patients with pancreatic cancer: the PancStress study

**DOI:** 10.1038/s41598-024-77010-8

**Published:** 2024-11-01

**Authors:** Alicia Sitte, Ruediger Goess, Tutku Tüfekçi, Ilaria Pergolini, Paulo Leonardo Pfitzinger, Eloísa Salvo-Romero, Carmen Mota Reyes, Sergey Tokalov, Okan Safak, Hendrik Steenfadt, Ibrahim H. Gürcinar, Ümmügülsüm Yurteri, Miriam Goebel-Stengel, Gemma Mazzuoli-Weber, Andreas Stengel, Mert Erkan, Helmut Friess, Rouzanna Istvanffy, Güralp Onur Ceyhan, Elke Demir, Ihsan Ekin Demir

**Affiliations:** 1 Department of Surgery, Klinikum rechts der Isar, School of Medicine, Technical University of Munich, Ismaninger Str. 22, 81675 Munich, Germany; 2 German Cancer Consortium (DKTK), Partner Site Munich, Munich, Germany; 3 CRC 1321 Modelling and Targeting Pancreatic Cancer, Munich, Germany; 4 Department of Surgery, Koc University School of Medicine, Istanbul, Turkey; 5 Laboratory of Neuro-Immuno-Gastroenterology, Digestive System Research Unit, Vall d’Hebron Institut de Recerca (VHIR), Vall d’Hebron Hospital Universitari, Barcelona, Spain; 6 Department of Internal Medicine, Psychosomatic Medicine and Psychotherapy, University Hospital Tübingen, Tübingen, Germany; 7 Department of Internal Medicine, Helios Klinik Rottweil, Rottweil, Germany; 8 University of Veterinary Medicine Hannover, Hannover, Germany; 9 Department for Psychosomatic Medicine, Charité Center for Internal Medicine and Dermatology, Charite - Universitätsmedizin Berlin, Corporate Member of Freie Universität Berlin, Humboldt-Universität zu Berlin and Berlin Institute of Health, Berlin, Germany; 10 HPB-Unit, Department of General Surgery, School of Medicine, Acibadem Mehmet Ali Aydinlar University, Istanbul, Turkey; 11 Else Kröner Clinician Scientist Professor for Translational Pancreatic Surgery, Munich, Germany; 12 Neural Influences in Cancer (NIC) International Research Consortium, Munich, Germany

**Keywords:** Anxiety, Depression, Distress, Mast cell, Pancreatic cancer, Pancreatic cancer, Tumour immunology, Surgical oncology, Quality of life, Cancer microenvironment

## Abstract

Mast cells are commonly found in pancreatic ductal adenocarcinoma (PDAC), yet their role in the disease remains uncertain. Although mast cells have been associated with depression in several diseases, their connection to PDAC in this context remains unclear. This study explored the correlation between mast cells and psychosocial stress in patients with PDAC. Prior to surgery, 40 patients with PDAC (n = 29 primary resected, n = 11 neoadjuvant treated) completed four questionnaires assessing stress and quality of life. Immunostaining was performed on the resected tumor tissue. Spearman analysis was employed to correlate mast cells with distress and neuropeptides serotonin and beta-endorphin serum and tissue levels. Patients with PDAC exhibited elevated levels of distress and worry. Lower number of mast cells within the tumor correlated with greater psychological burden. Among primary resected patients, mast cell count moderately correlated with joy and inversely with worries. Following neoadjuvant chemotherapy, strong inverse correlation was observed between anxiety, depression, and mast cell quantity. No correlation was found between mast cells and serotonin or beta-endorphin levels. In summary, mast cell presence inversely correlates with psychosocial stress, suggesting a link between immune cells and psychological well-being in pancreatic cancer. Targeting mast cells might offer therapeutic avenues for addressing cancer-induced depression and anxiety.

**Supplementary Information:**

The online version contains supplementary material available at 10.1038/s41598-024-77010-8.

## Introduction

Pancreatic carcinoma has a high mortality rate, with adenocarcinoma being the most common type (90%)^1^. The low 5-year survival rate of 11% for all stages is due to the advanced stage of the disease and metastasis at diagnosis. Only 15–20% of patients are candidates for curative surgical resection, with a 5-year survival rate of 40% ^2,3^. In 2018, Germany had 19,000 new cases of pancreatic cancer, and by 2030, it is projected to become the second leading cause of cancer-related deaths, with a 5-year survival rate of only 11%. Symptoms of pancreatic cancer include loss of appetite, steatorrhea, abdominal pain, and jaundice^4^. Psychological symptoms such as distress, depression, and anxiety occur in nearly 50% of cases before diagnosis^5,6^. Previous studies have found a correlation of depression and anxiety with mast cell accumulation, activation, and degranulation in patients with functional dyspepsia and mastocytosis^7,8^. However, studies that looked at the potential involvement of mast cells in the psychological stress of patients with cancer are lacking.

Mast cells contain various biologically active substances in their secretory granules and can produce neurotransmitters, such as beta-endorphin^9,10^. Mast cells also take up and release serotonin^11^. Degranulation can occur quickly or slowly^12^, and specific mediators, like serotonin, can be selectively secreted through degranulation-independent mechanisms^13^. Mast cell infiltration was shown to be increased in pancreatic ductal adenocarcinoma (PDAC) compared to healthy tissue, but its influence on the disease is not fully understood^14–16^. Previous studies assigned mast cells positive and negative or neutral prognostic properties. For example, Wang et al.^17^ showed a favorable influence of CD117 + mast cells on disease progression, while Hart et al.^18^ found no effect of tumoral mast cell count on overall survival. In contrast, others identified mast cells as a critical component in PDAC^15,19,20^. High mast cell numbers correlated with microvessel density, lymph node metastasis, and reduced survival. The zonal distribution of mast cells also plays a crucial role. Cai et al. demonstrated that high cell numbers in the intratumoral border zone were associated with advanced stage and increased metastasis^21^. Mast cell accumulation was most pronounced in the invasive edge zone, and intact mast cells were identified in PDAC, suggesting selective mediator release.

Distress, depression, and anxiety are common conditions/comorbidities in pancreatic cancer patients and can significantly reduce the quality of life^5^. Yaskin et al. described depression and anxiety as primary symptoms of pancreatic cancer back in the 1930s^22^. Patients with pancreatic cancer have the highest prevalence of psychological symptoms compared to other types of cancer^23^. The relationship between psychosocial stress and mast cell accumulation, activation, and degranulation in pancreatic cancer patients has not yet been studied.

For this reason, the PancStress study aimed to analyze the association between mast cell accumulation and activation and psychosocial burden in patients with PDAC. The study also compared individual psychosocial features and health-related quality of life of patients with PDAC exposed to different treatment settings, i.e., primary surgical resection vs. neoadjuvant treatment.

## Methods

The PancStress study was conducted as a prospective exploratory analysis on 40 patients with suspected PDAC who were candidates for surgery due to primary resectable PDAC or after neoadjuvant therapy for locally advanced PDAC. The patients (n = 29 with primary resectable and n = 11 with neoadjuvant-treated PDAC) were recruited from November 2018 to October 2022 at the Department of Surgery at the Klinikum rechts der Isar, Technical University of Munich, Germany. Ethical approval was obtained from the Ethics Committee of the Technical University of Munich (Reference number: 2018-333-S-KK). The study has been performed in accordance with the Declaration of Helsinki. Inclusion criteria were age over 18, radiological, clinical, or serological suspicion of PDAC, and written informed consent. Exclusion criteria included known mast cell-associated diseases, known synchronous or metachronous second cancers within five years before suspicion of PDAC, immunocompromised patients or those taking immunomodulatory drugs, patients with inherited or acquired immune system disorders, mental illnesses such as depression or anxiety disorder, patients with acute or chronic inflammation, inability to consent, and previous or simultaneous psychological, psychiatric, or psychosomatic therapy. Patients whose final histology showed no evidence of PDAC were subsequently excluded to not bias the results.

### Study procedure

**Fig. 1:**
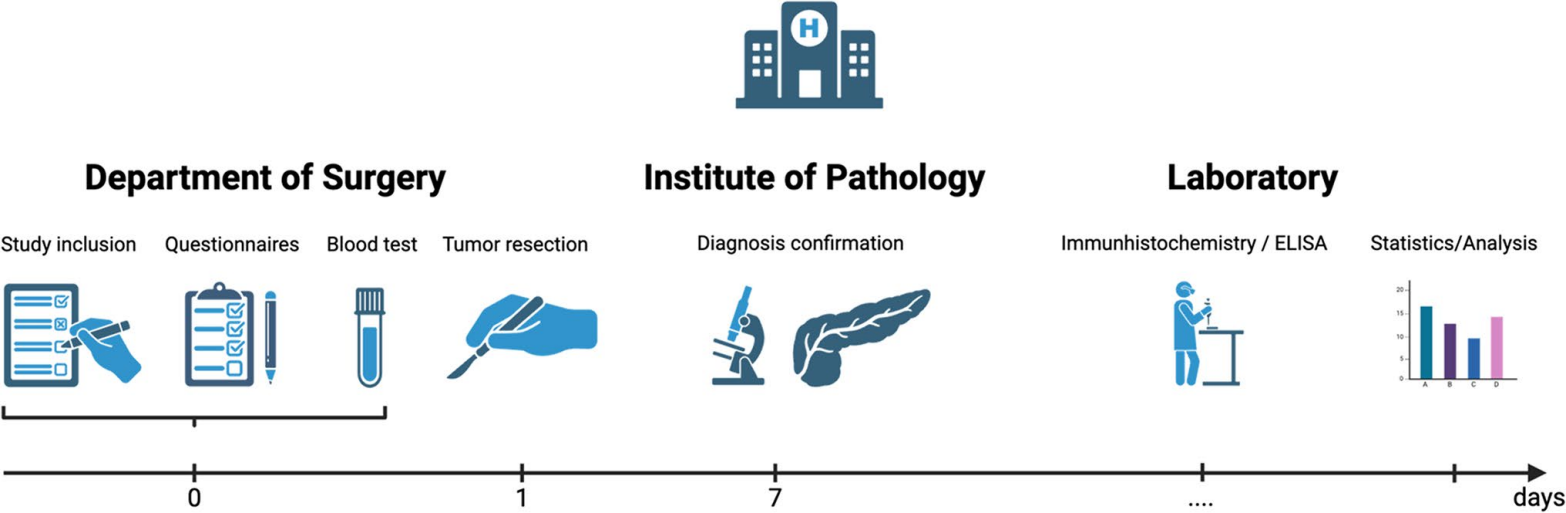
Graphical overview of the study work flow.

Patients who provided written consent were enrolled in the study. On the day before surgery, patients were given four validated and standardized questionnaires to assess their psychosocial burden. After surgery, histological diagnosis was confirmed, and histological specimens were obtained from the Department of Pathology. The tissue was fixed in 4% paraformaldehyde and embedded in paraffin. Questionnaire responses and histopathological stainings were evaluated. The automated quantification of mast cells was conducted on up to two tissue sections per patient (see results for details) using the QuPath software version 0.2.3 (Queens’s University of Belfast, Belfast, Northern Ireland, https://qupath.github.io/). Patient data were retrieved from the digitalized patient records in the hospital’s SAP system. A graphical overview of the study is shown in Fig. 1.

### Questionnaires

#### Distress thermometer

The Distress Thermometer is a screening tool designed by the National Comprehensive Cancer Network in the United States for patients with oncological diseases. It enables rapid identification of psychosocial stress, allowing for prompt initiation of treatment and care by other disciplines such as social services, psychological institutions, chaplaincy, or oncological institutions. It includes an 11-point Likert scale and asks about the past week, with scores ranging from 0 to 10. The questionnaire also lists several reasons that can lead to increased stress, such as family, emotional, religious, and physical issues. A score of 5 or higher is considered relevant distress^24^.

#### Hospital anxiety and depression scale (HADS)

HADS is a widely used self-assessment questionnaire to measure anxiety and depression in individuals aged 15 years and older. This questionnaire is helpful for quickly screening for psychological diseases associated with physical illnesses in routine clinical practice. The HADS questionnaire comprises seven questions related to anxiety and another seven related to depressive behavior^25^. A HADS score of ≤ 7 is considered normal, scores between 8 and 10 are borderline, and scores ≥ 11 indicate significant symptoms of anxiety and depression^26^.

#### Perceived stress questionnaire 20 (PSQ-20)

The PSQ-20 was developed for psychosomatic stress research to assess and measure subjective stress experience in adults^27^. It comprises 20 items, with five assigned to each factor: worries, tension, joy, and demands. The first three factors focus on internal stress reactions, while the demands scale evaluates environmental stressors. The worry scale addresses feelings of despair and future anxiety, while the tension scale assesses exhaustion, relaxation problems, and mental and physical tension. The joy scale encompasses energy, positive challenges, and feelings of security and is distinguished from the other scales by its positive formulation. Lastly, the demands scale addresses overwhelm, overwork, time pressure, and lack of time^28^. As part of the analyses, we compared the values of the PDAC cohort with a healthy adult sample. The healthy adult sample (n = 334) was taken from the survey results of a validation and reference study by Fliege et al.^28^. The healthy cohort comprised 61.6% female and 38.4% male patients with a median age of 45.3 years (18–88 years).

#### Short form 36 health survey (SF-36)

The SF-36 evaluation was manually performed using Bullinger and Kirchberger’s manual^29^. Raw scale values were modified for ten items and then converted to values between 0 and 100 using a formula in the manual. High values indicate higher quality of life, and low values indicate decreased quality of life from the participant’s perspective. Finally, the values were compared with the overall German norm sample from a representative population sample of 1994 (n = 2,914)^29^.

### Immunohistology

Tissue sections were initially deparaffinized with Roticlear (Carl Roth GmbH, Karlsruhe, Germany) substitute for 3 × 10 minutes. Subsequently, rehydration was performed using ethanol in descending alcohol series. In the next step, the specimens were washed with distilled water for 2 × 5 minutes and then in tris-buffered saline solution with Tween20 (TBST buffer) for five minutes. Next, the tissue samples were boiled for three minutes at a temperature of 600 °C and then for ten minutes at a temperature of 90 °C in citrate buffer (pH 6.0). Cooling was performed for 20 minutes at room temperature, followed by another five-minute cleaning in TBST buffer. Subsequently, the tissue samples were incubated in a humid chamber with 0.5% Triton X-100 (Carl Roth GmbH, Karlsruhe, Germany) phosphate-buffered saline (PBS) and 3% hydrogen peroxide (Carl Roth GmbH, Karlsruhe, Germany) for ten minutes each. A five-minute TBST buffer wash was performed between these two steps. After the hydrogen peroxide incubation, another five-minute wash with distilled water was performed. Finally, the Serum Free Protein Block “Ready-To-Use” from Dako GmbH (Jena, Germany) was applied to inhibit other antigen proteins, and the sections were stored at room temperature in a humid chamber for ten minutes. Then, the primary antibody Anti-Mast Cell Tryptase Antibody (1:10 000, Ab 2378, mouse monoclonal, Abcam, Cambridge, United Kingdom) or Anti-CD45 Antibody (1:2000, Ab 281586, rabbit multiclonal, Abcam, Cambridge, United Kingdom) was applied and incubated overnight in the refrigerator at 4 °C. After a 3 × 10-minute cleaning with TBST buffer the next day, the secondary antibody EnVision + System-HRP Labelled Polymer Anti-mouse or Anti-rabbit (Dako GmbH, Jena, Germany) was applied to the slides to detect the primary antibodies. The incubation time in the humid chamber at room temperature was one hour. After incubation, the tissue sections were rewashed with TBST buffer for 3 × 10 minutes and stained with 3,3’-diaminobenzidine chromogen solution (DAB) under the microscope. Finally, the sections were washed in distilled water for five minutes, and a counterstaining with hematoxylin (seven seconds per slide) was performed. As a further step, dehydration was performed in an ascending alcohol series. Finally, the sections were cleaned with Roticlear (3 × 5 minutes), and Menzel cover glasses (24 × 32 mm) were fixed onto the specimens with permanent mounting medium (Vecta Mount permanent Mounting Medium, Vector laboratories, Newark, USA).

### Quantification of mast cells and CD45-positive cells in primary tumor tissue sections

After immunohistochemical staining, the preparations were digitally scanned using the AxioScan.Z1 from Zeiss. The resulting images were evaluated using QuPath version 0.2.3 software (Queens’s University of Belfast, Belfast, Northern Ireland, https://qupath.github.io/). To measure the specific intratumoral mast cell count and the amount of CD45-positive cells, the whole tumor area on each slide was manually selected using the annotation tool of QuPath software, which obviated the need for high-power field selection or separate tumor areas. The “positive cell detection” feature automatically calculated the amount of positively stained cells in the selected tumor area. QuPath software was first trained with some positive stained cells, which the investigator selected. From the total number of positive cells in the selected tumor area, the number of positive cells per mm^2^ was calculated and used for further analysis. A representative staining with quantification of mast cell count is shown in Supplementary Fig. 1.

### Beta-endorphin and serotonin measurement

The neuropeptide levels of beta-endorphin and serotonin were measured in the serum and tissue of Patients via the Human Beta-Endorphin ELISA Kit (Wuhan EIAab Science, Cat. No: E0806h) and the Serotonin ELISA Kit (Abcam, Cat. No.: ab133053) according to the manufacturer’s instructions. The blood sample was obtained during routine blood collection on the day before the surgery after written informed consent. The serum was centrifuged at 1,000 x g for 15 min at room temperature, and the supernatant was stored at -80° until use. The tissue was obtained directly after tumor resection, frozen in liquid nitrogen, and stored at -80°. 0.05 g of the tissue was dissolved in 1 ml phosphate-buffered saline (PBS) and used for the analysis. All experiments were performed in triplicate (serum) or duplicate (tissue).

### Statistics

Microsoft Excel (Version 2301, Microsoft, Redmond, USA), GraphPad Prism 9 (Version 9.4.1, GraphPad Software, San Diego, USA), and IBM SPSS Statistics (Version 29.0, IBM, Armonk, USA) were used for data collection, statistical analysis, and graph creation. The collected data were checked for normal distribution using the D’Agostino & Pearson test. If the datasets had a Gaussian distribution, the t-test for independent samples was used for comparison. Otherwise, the Mann-Whitney test was applied. The Spearman rank correlation coefficient was used for the main research question, where the number of positive mast cells per mm^2^ was related to various individual scores from SF-36 and PSQ-20, a total index value of PSQ-20, and scores of the Distress Thermometer and two subscales of HADS. Z-value as standard deviations from the mean were used to compare the results of SF-36 with a German norm cohort as described before^29^. As the PancStress study was exploratory, the resulting correlation coefficient was considered hypothesis-generating, not confirmatory. All results were analyzed and reported with a significance level of *p* < 0.05. The results were shown with ± standard deviation (SD). The confidence interval was 95%.

## Results

### Patient characteristics

The PancStress study enrolled 64 participants, with 40 patients meeting the criteria for final analyses. Twenty-four patients were excluded from the final analysis due to various reasons, including lack of pancreatic ductal adenocarcinoma on definitive histology (n = 9), inoperability or unavailability of tissue (n = 6), chronic inflammation (n = 1), synchronous or metachronous tumor within the last five years (n = 3), mast cell-associated diseases (n = 1), or pre-existing mental illnesses (n = 4). The participant’s ages ranged from 41 to 87 years, with an average age of 67.2 years at the time of surgery. Among them, 29 patients underwent primary tumor resection, while 11 patients received neoadjuvant chemotherapy for locally advanced tumors. Patient and tumor characteristics are summarized in Table 1.

**Table 1:** Patient and tumor characteristics.

Parameter	Total	Upfront surgery	Neoadjuvant chemotherapy	*p*-value^†^
	*n = 40 (%)*	*n = 29 (%)*	*n = 11 (%)*	
Sex				
Female	23 (57.5)	16 (55.2)	7 (63.6)	0.73
Male	17 (42.5)	13 (44.8)	4 (36.4)	
Tumour localization				
Head	32 (80)	23 (79.3)	9 (81.8)	1
Body	4 (10)	2 (6.9)	2 (18.2)	
Tail	4 (10)	4 (13.8)	0 (0)	
Type of resection				
Pancreaticoduodenectomy	24 (60)	18 (62.1)	6 (54.5)	0.88
Distal pancreatectomy	8 (20)	6 (20.7)	2 (18.2)	
Total pancreatectomy	8 (20)	5 (17.2)	3 (27.3)	
T stage (AJCC, 8th edition)				
T1	1 (2.5)	1 (3.4)	0 (0)	< 0.001*
T2	28 (70)	20 (69.0)	8 (72.7)	
T3	10 (25)	8 (27.6)	2 (18.2)	
T4	1 (2.5)	0 (0)	1 (9.1)	
Lymph node status				
N0	12 (30)	7 (24.2)	5 (45.4)	0.18
N1	16 (40)	11 (37.9)	5 (45.4)	
N2	12 (30)	11 (37.9)	1 (9.2)	
UICC stadium classification				
IA	1 (2.5)	1 (3.4)	0 (0)	0.12**
IB	7 (17.5)	3 (10.3)	4 (36.4)	
IIA	4 (10)	3 (10.3)	1 (9.1)	
IIB	15 (37.5)	10 (34.5)	5 (45.5)	
III	12 (30)	11 (37.9)	1 (9.1)	
IV	1 (2.5)	1 (3.4)	0 (0)	
Tumour grading				
1	1 (3.5)	1 (3.4)	n.a.	
2	20 (69)	20 (69.0)	n.a.	
3	8 (27.5)	8 (27.6)	n.a.	
4	0 (0)	0 (0)	n.a.	

n.a.: not analysed; ^†^Fisher test; *T1/2 vs. T3/4; **IA-IIA vs. IIB vs. III/IV.

### Available questionnaires

**Fig. 2:**
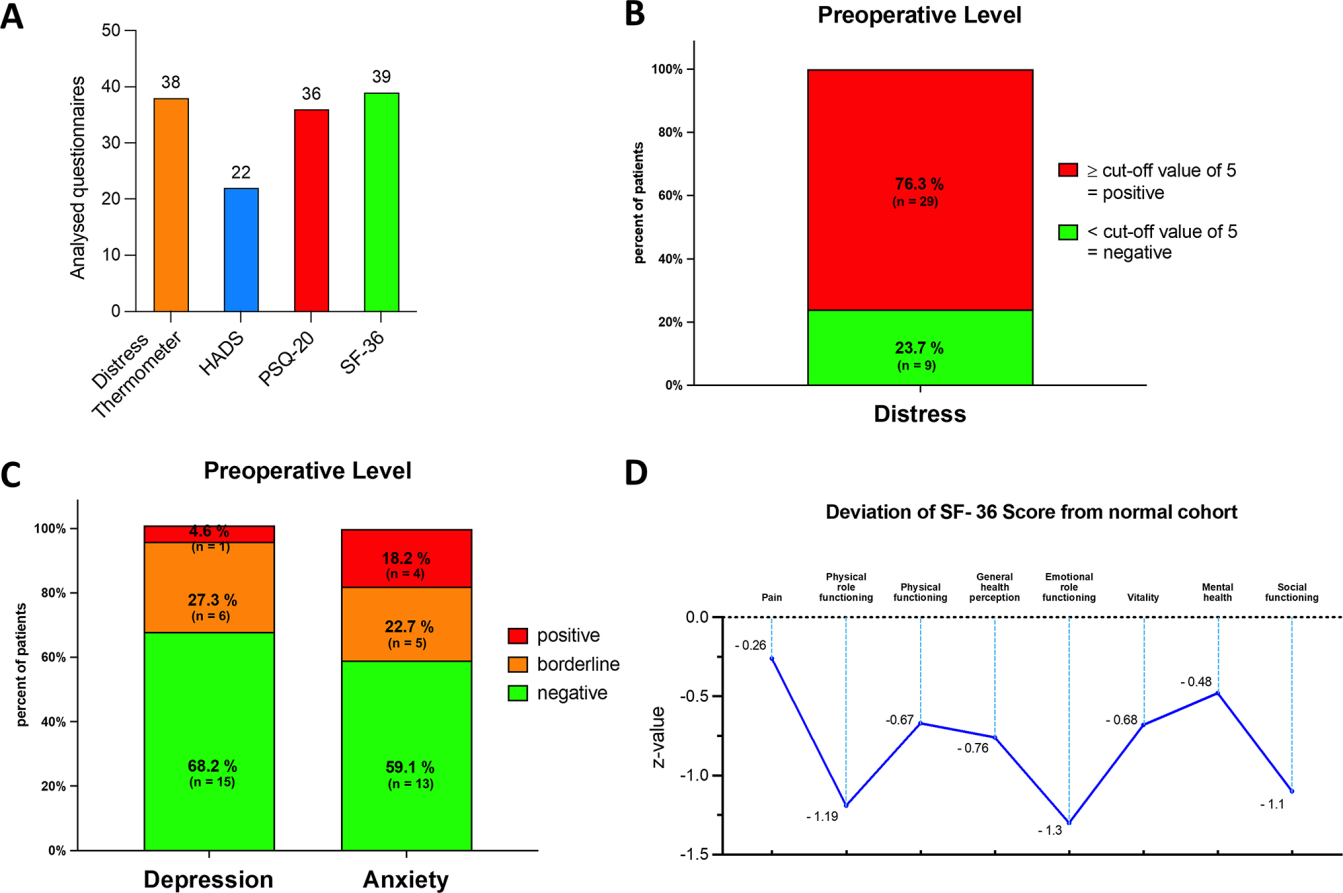
Overview of all analyzed questionnaires (**A**) preoperative levels of distress using the distress thermometer (**B**) and depression and anxiety using the hospital anxiety and depression scale (HADS) questionnaire (**C**) deviation of SF-36 results compared to the normal cohort using z-values (**D**).

The Distress thermometer, HADS, PSQ-20, and SF-36 were used to assess psychosocial burden. Figure 2A provides an overview of the number of available questionnaires.

### Results of the distress thermometer

The Distress thermometer was used to measure the emotional distress levels of patients, with a cutoff value of ≥ 5 ^24^. Emotional problems were identified as the most important factor contributing to increased distress levels, with anxiety, worry, nervousness, sadness, and loss of interest in everyday activities being the most prevalent (Table 2). Preoperative emotional distress was observed in 76.3% of patients (Fig. 2B). The distress level values between patients who underwent upfront surgery (US) and those who underwent resection after neoadjuvant chemotherapy (NCT) did not show any significant difference (6.4 ± 2.8 vs. 5.0 ± 2.3, *p* = 0.1843). The age and gender of the patients had no impact on the value of distress level (Supplementary Table 1, male vs. female *p* = 0.28).

**Table 2:** Categorization of problems based on the distress thermometer.

	Yes (*n*)	Analyzed questionnaire	Rate (%)
Practical problems			
Housing	1	35	2.9
Insurance	2	35	5.7
Work/school	4	33	12.1
Transportation	4	35	11.4
Childcare	0	33	0
Financal worries	4	35	11.4
Caregiving for family members	4	32	12.5
Total	19	238	8
Family problems			
Dealing with partner	0	34	0
Dealing with children	0	34	0
Total	0	34	0
Emotional problems			
Worries	20	37	54.1
Anxiety	21	36	58.3
Sadness	14	37	37.8
Depression	6	36	16.7
Nervousness	14	36	38.9
Loss of interest in daily activies	9	36	25
Total	87	218	39.9
Spiritual/religious problems			
Regarding God	2	34	5.9
Loss of faith	2	34	5.9
Total	4	68	5.9
Physical problems			
Pain	16	36	44.4
Nausea	8	36	22.2
Fatigue	17	37	45.9
Sleep	17	35	48.6
Movement/Mobility	11	35	31.4
Washing/Dressing	2	35	5.7
Appearance	3	34	8.8
Breathing	4	35	11.4
Mouth sores/inflammation	3	36	8.3
Eating/Nutrition	8	34	23.5
Digestive Problems	19	37	51.4
Constipation	9	36	25
Diarrhea	18	37	48.6
Urination changes	8	35	22.9
Fever	3	35	8.6
Dry/itchy Skin	13	35	37.1
Dry/stuffy nose	7	36	19.4
Tingeling in hand/feet	13	37	35.1
Feeling swollen/bloated	5	36	13.9
Hot flashes/sweating	9	36	25
Dizziness	4	36	11.1
Memory/Concentration	9	36	25
Sexual problems	0	34	0
Total	206	819	25.2

### Results of the hospital anxiety and depression scale

Of the 22 patients with PDAC before surgery, seven patients (31.9%) had elevated scores on the depression scale, with six patients (27.3%) being borderline and one patient (4.6%) showing significant depression. 13 (59.1%) of the 22 patients had anxiety symptoms before surgery, with five patients (22.7%) having borderline and four patients (18.2%) having significant anxiety scores (Fig. 2C). Patients who received NCT had significantly higher scores (3.5 ± 3.1 vs. 8.0 ± 3.4, *p* = 0.0196) on the depression scale preoperatively. There was no significant difference in anxiety between the two groups (5.9 ± 3.7 vs. 8.8 ± 2.6, *p* = 0.1083). Anxiety and depression scales were not dependent on the age and gender of the patients (Supplementary Table 2, male vs. female: anxiety scale: *p* = 0.73, depression scale: *p* = 0.52).

### Results of the perceived stress questionnaire 20

The PDAC cohort was compared with a healthy adult cohort using PSQ-20. Although only slight differences were observed in tension, worries, and joy domains, they were not statistically significant (p-values were *p* = 0.802, *p* = 0.548, and *p* = 0.328, respectively). However, the healthy adult cohort had significantly higher results in the demands category (*p* = 0.005), which led to an increased overall stress level (*p* = 0.752) (Table 3). No significant differences were found when comparing PSQ-20 results between US and NCT patients. However, there was a tendency towards significance in the joy category, with NCT patients having a lower scale value (NCT: 45.2 ± 22.4 vs. US: 62.0 ± 23.7, *p* = 0.079).

**Table 3:** Comparison of PSQ-20 result between PDAC patients and healthy control.

	PDAC	Healthy control	*p*-value^†^
	n = 36	n = 334	
Worries	28.4 ± 21.9*	26 ± 20	0.538
Tension	35 ± 22,7	34 ± 21	0.802
Joy	57.8 ± 24.5	62 ± 21	0.328
Demands	25.4 ± 20.1	36 ± 21	0.005
Overall experience of stress	32 ± 17.8*	33 ± 17	0.752

Data are shown in mean ± SD, ^†^Mann Whitney test, *n = 35.

### Results of the short form 36 health survey

Patients with PDAC rated their health status worse than the German norm cohort across all areas of life (Table 4). Highly significantly lower values were found in all categories except pain (71.9 ± 28.0 vs. 79.1 ± 27.4, *p* = 0.136). Reference values for the German norm cohort were obtained from the manual by Bullinger and Kirchberger^29^, and Z-scores were calculated for a more precise comparison. The largest deviations from the German norm sample were observed in physical role functioning (-1.19), emotional role functioning (-1.3), and social functioning (-1.1) (Fig. 2D). No significant difference was detected in any subscale when comparing the quality of life between US and neoadjuvant NCT patient groups (Supplementary Table 2).

**Table 4:** Comparison of SF-36 subscales between PDAC patients and a German norm cohort.

	PDAC	German norm cohort	*p*-value^†^
Physical functioning	n = 38	n = 2886	
	70.8 ± 30.3	85.7 ± 22.1	< 0.001
Physical role functioning	n = 36	n = 2856	
	45.8 ± 4.5	83.7 ± 31.7	< 0.001
Pain	n = 39	n = 2905	
	71.9 ± 28.0	79.1 ± 27.4	0.136
General health perception	n = 38	n = 2859	
	52.8 ± 17.5	68.1 ± 20.2	< 0.001
Emotional role functioning	n = 38	n = 2855	
	57.0 ± 45.2	90.4 ± 25.6	< 0.001
Vitality	n = 39	n = 2876	
	50.8 ± 20.4	63.3 ± 18.5	< 0.001
Mental health	n = 39	n = 2871	
	66.1 ± 18.6	73.9 ± 16.4	< 0.001
Social functioning	n = 39	n = 2911	
	68.6 ± 31.8	88.8 ± 18.4	< 0.001

All data shown in mean ± SD, ^†^Mann-Whitney test.

### Number of detected mast cells in tumor tissue

**Fig. 3:**
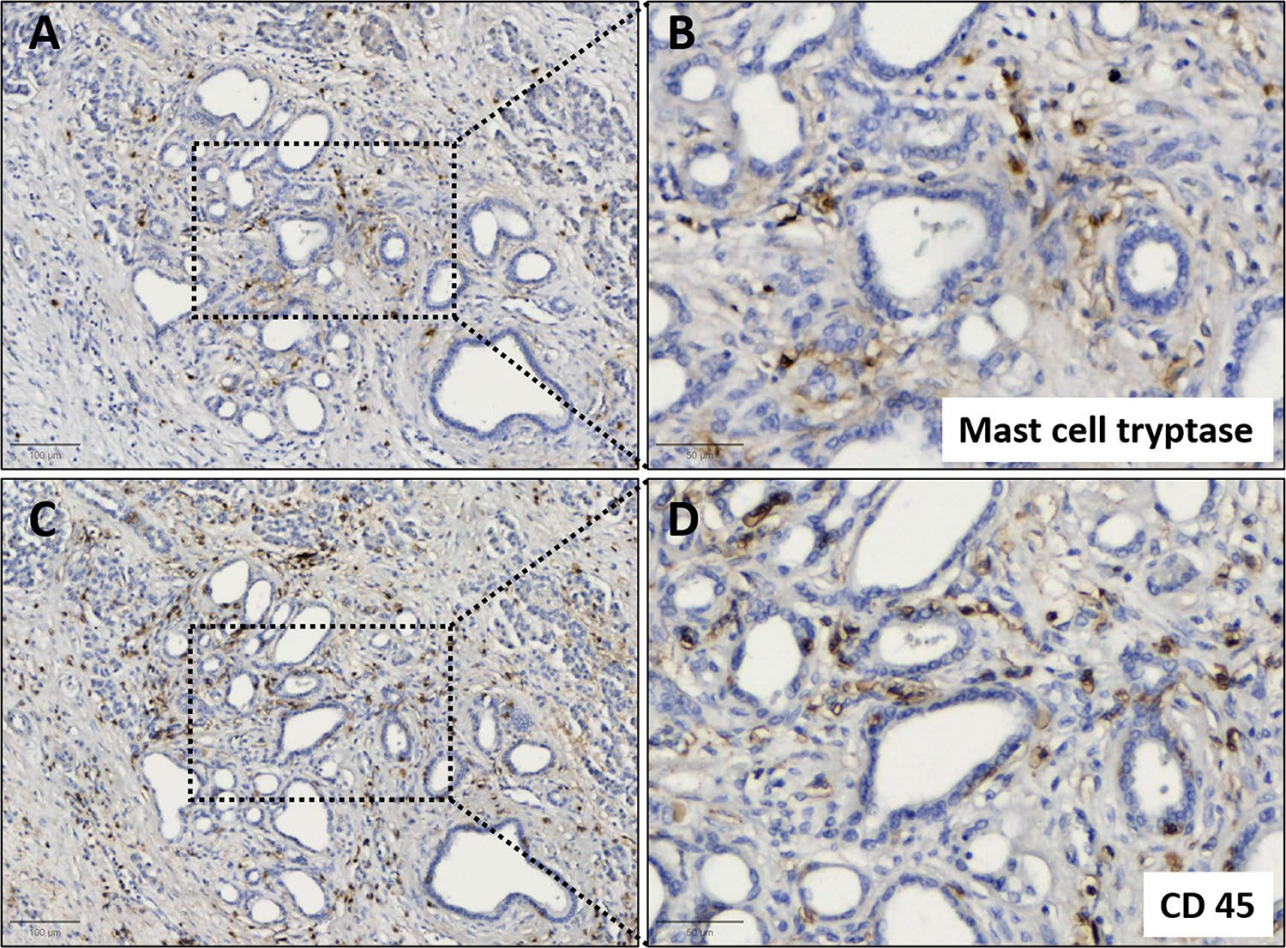
Example immunohistochemical staining of mast cell tryptase and CD45 with hematoxylin counterstaining on PDAC sample: (**A**) Mast cell tryptase, scale bar 100 μm (**B**) selected area with magnification 10x, scale bar 50 μm (**C**) CD 45, scale bar 100 μm (**D**) selected area with magnification 10x, scale bar 50 μm.

The analysis included a total of 58 tumor sections. Among these sections, 18 patients had two tumor sections evaluated, while the remaining 22 patients had only one due to limited tissue availability. The intratumoral mast cell count per mm^2^ for the total cohort (n = 40) was 49.74 ± 32.52 (% of all nucleated cells: 2.15 ± 1.30); for the US group (n = 29), it was 50.51 ± 36.10 (% of all nucleated cells: 1.97 ± 1.04), and for the NCT group (n = 11), it was 47.71 ± 21.66 (% of all nucleated cells: 2.52 ± 1.69) (*p* = 0.905). To evaluate the percentage of mast cells to the total amount of infiltrated immune cells, immunohistochemical staining of the pan-leukocyte marker CD45 was performed in the consecutive tumor sections in 33 patients (totaling 36 tumor sections). In these patients, the mean mast cell count per mm^2^ was 49.86 ± 34.14, and the amount of CD45 positive cells per mm^2^ was 796.20 ± 738.50. Therefore, 6.26% of all infiltrated (CD45 positive) immune cells were mast cells. An exemplary immunohistochemical staining of mast cells and pan-leukocyte marker CD45 is depicted in Fig. 3.

### Correlation of intratumoral mast cell count and distress, depression and anxiety level

Our study observed a correlation between intratumoral mast cell count and distress level, revealing that increased intratumoral mast cell count was associated with decreased distress levels. While this inverse correlation was moderate in the entire cohort (*r*=-0.3303, *p* = 0.043), it did not persist in the subgroups analyzed. However, a strong negative correlation was noted in the NCT group (n = 6) between intratumoral mast cell count and depression level (*r*=-0.8986, *p* = 0.028). No significant correlation was found in the US group or the overall cohort.

Similarly, a strong inverse correlation was observed in the NCT group between intratumoral mast cell count and anxiety level (*r*=-0.8827, *p* = 0.044), while no significant correlation was found in the US group or the overall cohort. These results are summarized in Table 5, and plots are provided in Supplementary Fig. 2.

**Table 5:** Correlation of mast cells count with distress, depression and anxiety levels.

	n	Spearmann-Rho	*p*-value
Distress level*			
Total cohort	38	− 0.3303	0.043
Upfront surgery	27	− 0.2755	0.164
Neoadjuvant chemotherapy	11	− 0.3797	0.251
Depression^†^			
Total cohort	22	0.09611	0.671
Upfront surgery	16	0.2206	0.409
Neoadjuvant chemotherapy	6	− 0.8986	0.028
Anxiety^†^			
Total cohort	22	− 0.1884	0.401
Upfront surgery	16	− 0.1616	0.547
Neoadjuvant chemotherapy	6	− 0.8827	0.044

*Distress thermometer; ^†^Hospital anxiety and depression Scale (HADS) questionnaire.

Additionally, the correlation between intratumoral mast cell count and levels of worry, tension, joy, demand, and overall stress experience are presented in Table 6 (with plots available in Supplementary Fig. 3). A moderate negative correlation was found between intratumoral mast cell count and the domain of worry, which was statistically significant in the overall cohort (*p* = 0.024) and the US group (*p* = 0.037). A similar association was observed in the tension domain (*p* = 0.079). Joy was positively correlated with intratumoral mast cell count, with a moderate effect size and statistical significance in the overall cohort (*p* = 0.032) and US group (*p* = 0.046). No significant relationships were found in the demand domain. Furthermore, a moderate negative correlation was observed between overall stress experience and intratumoral mast cell count in the overall cohort (*p* = 0.034), with a tendency towards significance in the US group (*p* = 0.065).

**Table 6:** Correlation of mast cells count with the four PSQ-20 subscales.

	n	Spearmann-Rho	*p*-value
Worries			
Total cohort	35	− 0.3808	0.024
Upfront surgery	26	− 0.4113	0.037
Neoadjuvant chemotherapy	9	− 0.3096	0.415
Tension			
Total cohort	36	− 0.2963	0.079
Upfront surgery	27	− 0.2556	0.198
Neoadjuvant chemotherapy	9	− 0.1604	0.68
Joy			
Total cohort	36	0.3584	0.032
Upfront surgery	27	0.3876	0.046
Neoadjuvant chemotherapy	9	0.3766	0.317
Demands			
Total cohort	36	− 0.0797	0.644
Upfront surgery	27	− 0.1213	0.547
Neoadjuvant chemotherapy	9	0.1826	0.656
Overall experience of stress			
Total cohort	35	− 0.3595	0.034
Upfront surgery	26	− 0.3672	0.065
Neoadjuvant chemotherapy	9	− 0.1765	0.647

### Correlation of intratumoral mast cell count with health-related quality of life

There were no significant correlations between intratumoral mast cell count and the individual health-related quality of life subscales (Supplementary Table 3). However, it should be noted that a positive relationship (*r* = 0.2841) with a tendency towards significance (*p* = 0.08) was observed in the mental health category (Plots are shown in Supplementary Fig. 4).

### Correlation of intratumoral mast cell count with clinical characteristics

The mast cell count of each patient was correlated with clinical characteristics and relevant blood values taken on the day of study inclusion. However, there was no statistically significant correlation of the mast cell count with the body mass index, the status of diabetes mellitus, smoking history, hypertension, jaundice, and the blood value of CA 19 − 9, leukocytes, hemoglobin, creatinine, albumin, and bilirubin (Supplementary Table 4).

### Correlation of intratumoral mast cell count with serotonin and beta-endorphin serum and tissue levels

Blood samples from five and frozen tissue of six pancreatic cancer patients were subjected to ELISA analysis to measure serotonin and beta-endorphin levels. The mean concentration of serum serotonin was 144.8 ± 83.8 ng/ml, while serum beta-endorphin was 4.9 ± 0.04 ng/ml. However, there was no significant correlation observed between intratumoral mast cell count and serum serotonin level (*r*=-0.2928, *p* = 0.63) or serum beta-endorphin level (*r*= -0.0718, *p* = 0.88). Interestingly, the serum serotonin levels in patients exhibited considerable fluctuations, whereas serum beta-endorphin levels remained remarkably stable (Supplementary Fig. 5A + B). In tissue, the mean concentration of serotonin was 0.028 ± 0.020 ng/ml, while the level of beta-endorphin was 0.70 ± 1.0 ng/ml. No significant correlation was observed between intratumoral mast cell count and serotonin tissue level (*r*=-0.3762, *p* = 0.46) or beta-endorphin tissue level (*r* = 0.4885, *p* = 0.33) (Supplementary Fig. 5C + D).

## Discussion

The PancStress study investigated psychosocial stress and mast cell infiltration in patients with PDAC for the first time, revealing an unexpected inverse correlation between mast cell count and distress. The study showed that higher mast cell counts were associated with better psychological well-being and less stress levels. Despite this correlation, patients with PDAC still experienced heightened emotional stress and diminished quality of life compared to healthy controls.

PDAC exhibits the highest prevalence of distress, depression, and anxiety among various cancers, particularly in the preoperative setting^30–32^. According to the findings from the PancStress study, 76.3% of patients experienced elevated distress levels, primarily driven by anxiety and worries, aligning with previous research^5,33–35^. Anxiety and emotional distress often escalate in PDAC patients awaiting surgery, fueled by uncertainties regarding surgical outcomes, concerns of physical complications, and the overarching fear of mortality^36,37^. Depressive symptoms were also prevalent in 31.9% of PDAC patients in the PancStress study, consistent with reported rates by Clark et al.^5^, Janda et al.^38^, and Kelsen et al.^39^, ranging from 28.8 to 38%. Similarly, anxiety symptoms were widespread in the current cohort, with rates of 40.9% according to the HADS questionnaire and 58.3% based on the Distress Thermometer, although Janda et al. reported lower clinically relevant anxiety rates (28%) and Clark et al. reported rates of 29.1%. The fear of surgery and preoperative anxiety contribute significantly to heightened anxiety levels in PDAC patients^40,41^. These findings underscore the critical need to address the psychological aspects accompanying PDAC treatment. Understanding the prevalence and severity of these factors can guide the development of effective interventions aimed at improving patient’s quality of life.

This study revealed only slight differences in scale values between patients with PDAC and healthy adults, possibly due to age-related variations in coping strategies^42^. While the average age of our patients was 67.2 years, the age of the comparison group had an average age of 45.3 years. PDAC patients reported more internal stressors, although external stress factors such as work-related stress and caregiving demands were minimal. However, patients reported reduced joy and increased emotional tension, particularly in the weeks preceding surgery, consistent with previous research on the psychological state of PDAC patients. The difference in stress experiences can be attributed to the various assessment periods of the questionnaires.

The study found that patients with PDAC had a significantly worse health-related quality of life than the German control population, except for pain, with the most significant deviations in emotional and physical role function and social functioning. The COVID-19 pandemic may have influenced this result, as another study found that patients with PDAC had a decline in social functioning, emotional, and physical role function due to the pandemic^43^. Studies consistently demonstrate reduced quality of life in patients with PDAC compared to the general population, with poor physical and mental quality of life reported in pancreatic cancer patients in different measuring instruments^44,45^. However, the PancStress study is unique as all patients with PDAC received curative resection, providing more precise values for health-related quality of life in PDAC with curative treatment intent.

This study also compared the psychological constitution and quality of life in patients with PDAC who underwent resection with or without NCT. Patients who received NCT had higher levels of depression and lower levels of joy preoperatively compared to those who underwent upfront surgery. However, there were no significant differences in other categories. We found no significant differences in health-related quality of life between the two groups. Our results are consistent with previous studies and suggest that NCT does not significantly affect the overall quality of life of patients with PDAC^46,47^. However, existing literature on other oncological patients has shown that depression and anxiety values almost doubled during NCT^48,49^. In our study, the NCT group had significantly higher depression scores before surgery, highlighting the need to address psychological factors in this treatment setting.

The neurotransmitters and neurohormones levels, including serotonin, endorphin, and dopamine, seem essential in this context since they are closely related to emotional states^50^. Studies in the past have shown that serotonin^51,52^ and endorphin^53^ levels are associated with positive affect, mental well-being, and depression, while dopamine^50^ release is upregulated during positive affective states. Mast cells can uptake, store, and release serotonin and beta-endorphin, and beta-endorphins can also act externally on mast cells and induce the release of various biomolecules^9,54,55^. In contrast, dopamine is not naturally present in mast cells, but it can be taken up and stored in the granules and induce mast cell degranulation^56,57^. Therefore, it is hypothesized that endorphins and dopamine can stimulate mast cells, leading to their activation and degranulation. This activation of mast cells due to the increased circulation of these neurotransmitters could result in an increased accumulation and activation of mast cells in a better psychological state. Mast cells can also release mood-influencing neurotransmitters such as beta-endorphin and serotonin into the bloodstream, potentially affecting the psychological state. Analyzed serotonin and beta-endorphin serum and tissue levels revealed no evident correlation with the intratumoral mast cell count in our study. However, it should be mentioned that these data were based on a limited sample size of only five (serum) and six (tissue) patients. The beta-endorphin levels exhibited minimal variation despite substantial differences in the plasma serotonin levels, prompting further investigation in a larger cohort. Since the PancStress study is exploratory, these are only assumptions, and additional scientific studies are required to investigate this relationship.

Due to various constraints, this study’s statistical power is reduced. Factors such as a small sample size, potentially influenced by COVID-19 and the lengthy questionnaires, are significant contributors. Additionally, it’s noteworthy that the majority of tumors were located in the pancreatic head (80%). Studies have shown substantial differences in the immune cell distribution across different regions of the organ, which could affect the comparability between tumors versus those in the body or tail^58^. It also should be mentioned that only 1–2 tumor slides per patient were analyzed, which cannot reflect tissue heterogeneity.

In conclusion, our study has revealed an intriguing inverse correlation between intratumoral mast cells count and psychosocial stress in PDAC patients. We found that fewer mast cells were associated with higher levels of distress, worries, and overall perceived stress, while increased levels of joy correlated with more mast cells. This novel discovery highlights the need for further investigations into the complex relationship between mast cell enrichment and psychosocial burden in PDAC. Understanding the interplay between mast cells and psychological factors holds promise for developing innovative treatment approaches to enhance the mental well-being of PDAC patients.

## Electronic supplementary material

Below is the link to the electronic supplementary material.

Supplementary Material 1

## Data Availability

All data are available from the Corresponding Author upon reasonable request.
