# Supplementary material for: Correlation of intratumoral mast cell quantity with psychosocial distress in patients with pancreatic cancer: the PancStress study
Source: Sci Rep. 2024 Nov 1;14:26285. doi: 10.1038/s41598-024-77010-8 (PMC11530627; doi:10.1038/s41598-024-77010-8)
Supplement: Supplementary file 1 — Supplementary Material 1 [file 41598_2024_77010_MOESM1_ESM.docx]

## Supplementary Figure 1

**
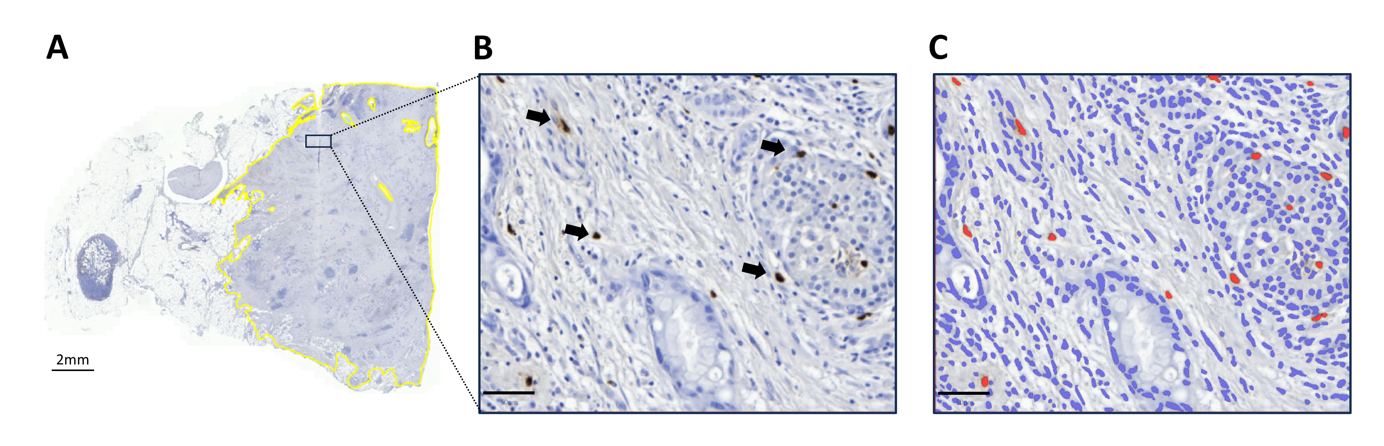
**

**Supplementary Figure 1:** Representative immunohistochemical mast cell tryptase staining of PDAC with HE counterstaining and the performed analysis using QuPath software. (A) whole slide with selected and analyzed tumor area in yellow (B) Quantification of the marked area with positive stained cells (black arrows) (B) Positive cell detection in red and negative cells in blue using QuPath software. Scale bar: 50μm.

## Supplementary Figure 2


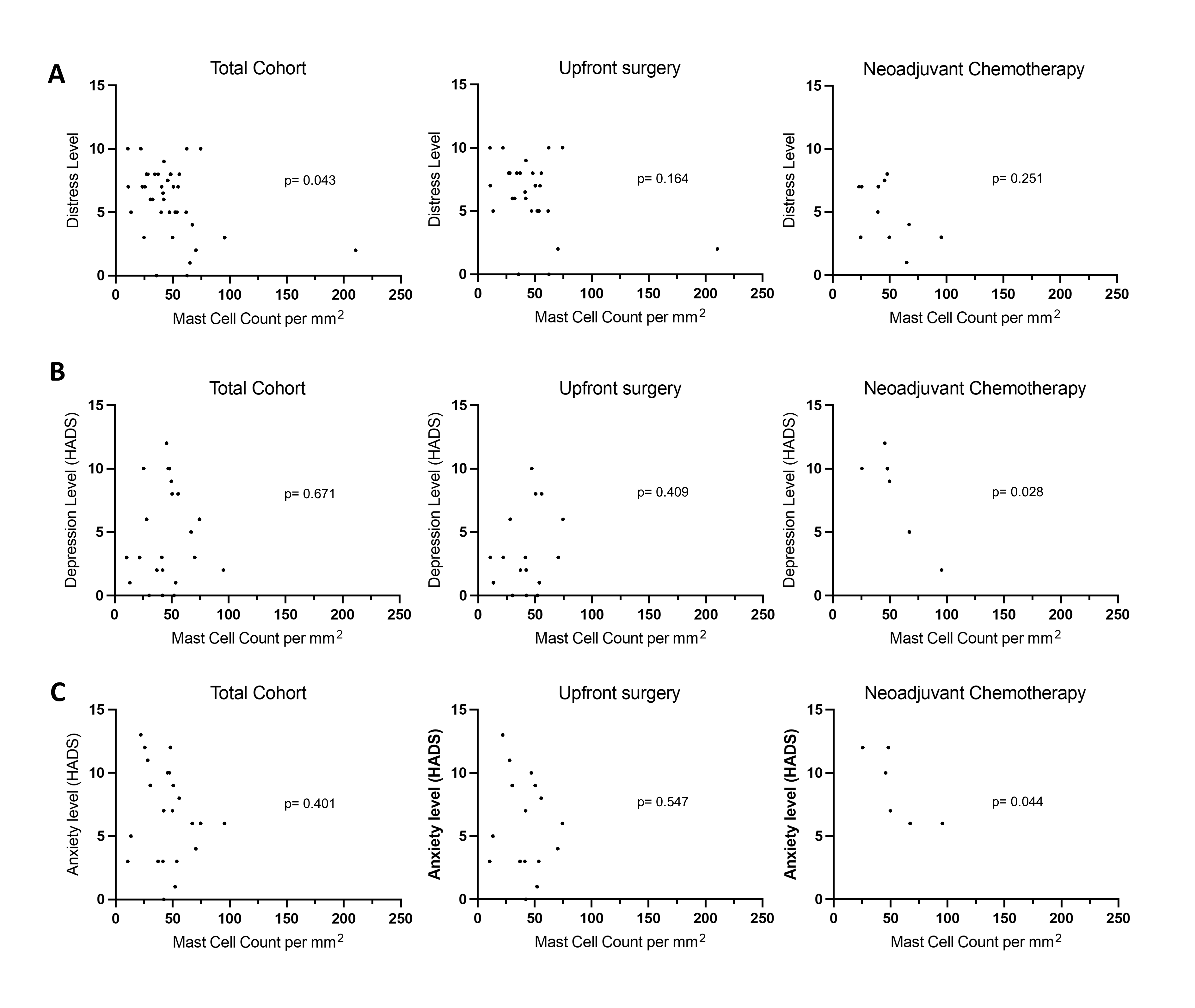


**Supplementary Figure 2:** Correlation of mast cell count with (A) Distress using the Distress thermometer and with (B) Depression Level and (C) Anxiety Level using the Hospital Anxiety and Depression Scale (HADS) questionnaire. The Spearman rank correlation coefficient was used for statistical analysis.

## Supplementary Figure 3


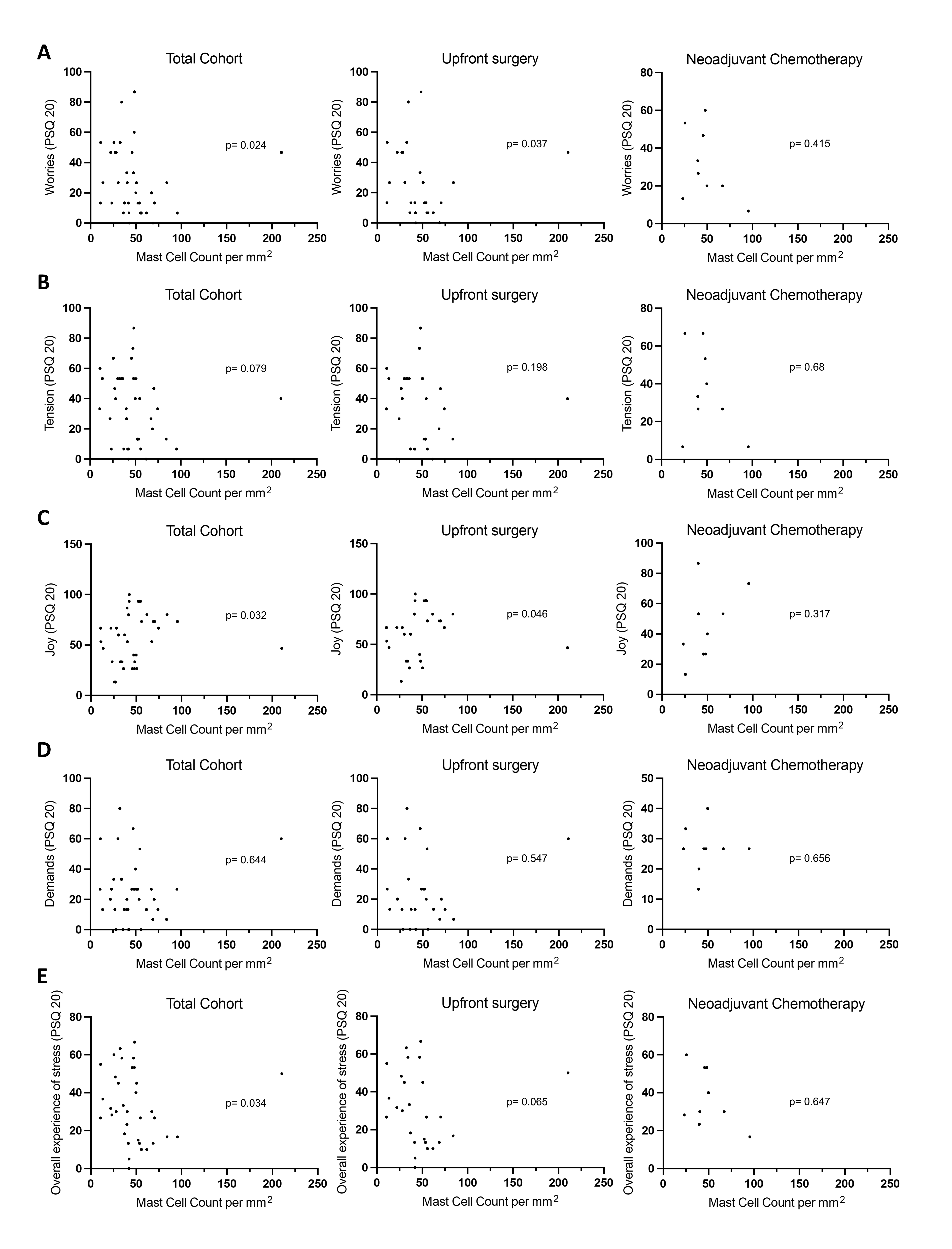


**Supplementary Figure 3:** Correlation of mast cell count with (A) Worries, (B) Tension, (C) Joy, (D) Demands, and (E) Overall experience of stress using the PSQ 20 questionnaire. The Spearman rank correlation coefficient was used for statistical analysis.

## Supplementary Figure 4


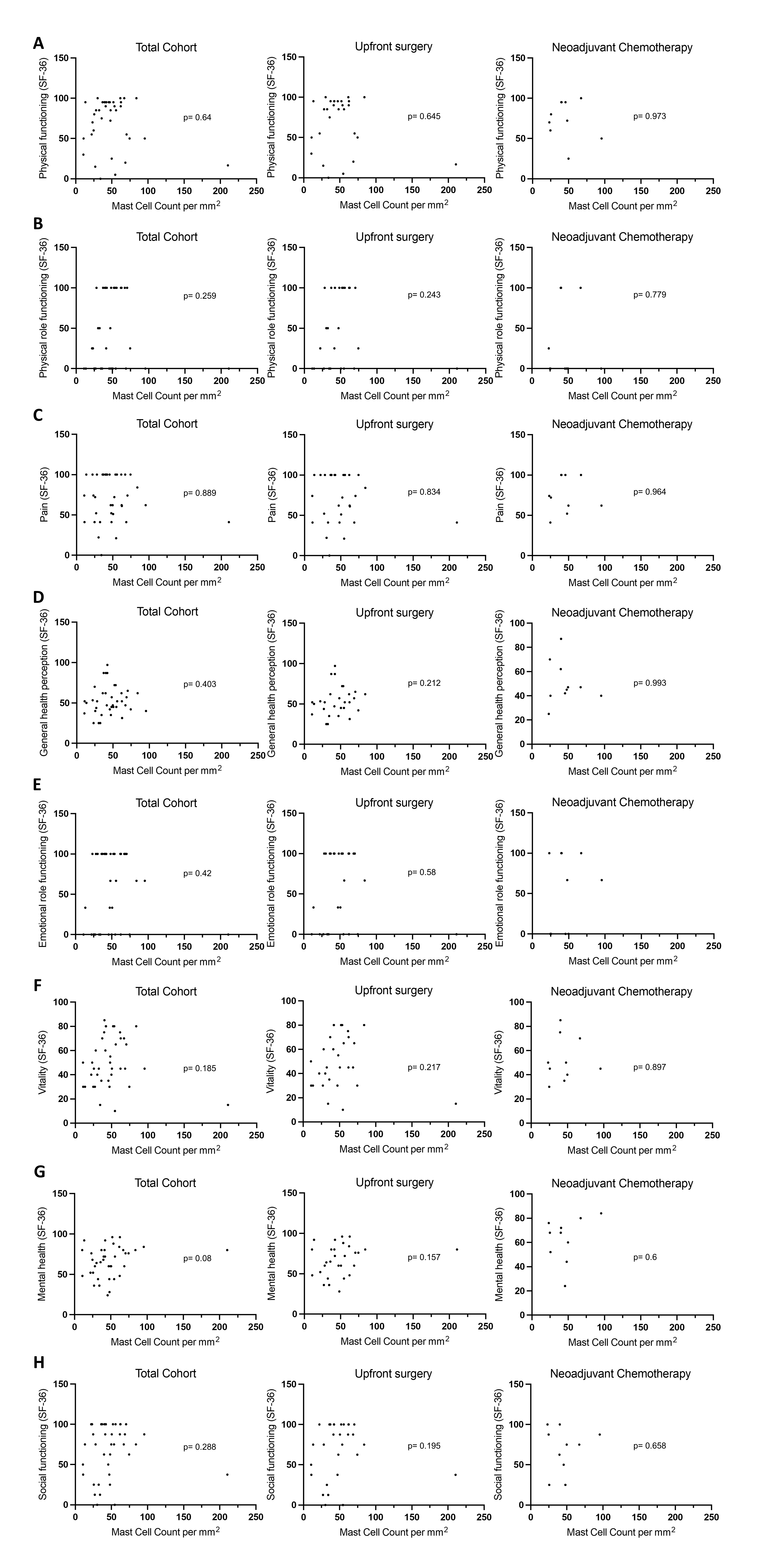


**Supplementary Figure 4:** Correlation of mast cell count with health-related quality of life: (A) Physical functioning, (B) Physical role functioning, (C) Pain, (D) General health perception, (E) Emotional role functioning, (F) Vitality, (G) Mental health, (H) Social functioning using the SF-36 questionnaire. The Spearman rank correlation coefficient was used for statistical analysis.

## Supplementary Figure 5

**Supplementary Figure 5:** Correlation of mast cell count with the serotonin serum level (A), the beta-endorphin serum level (B), the serotonin tissue level (C) and the beta-endorphin tissue level (D), serum level n=5, tissue level n=6.


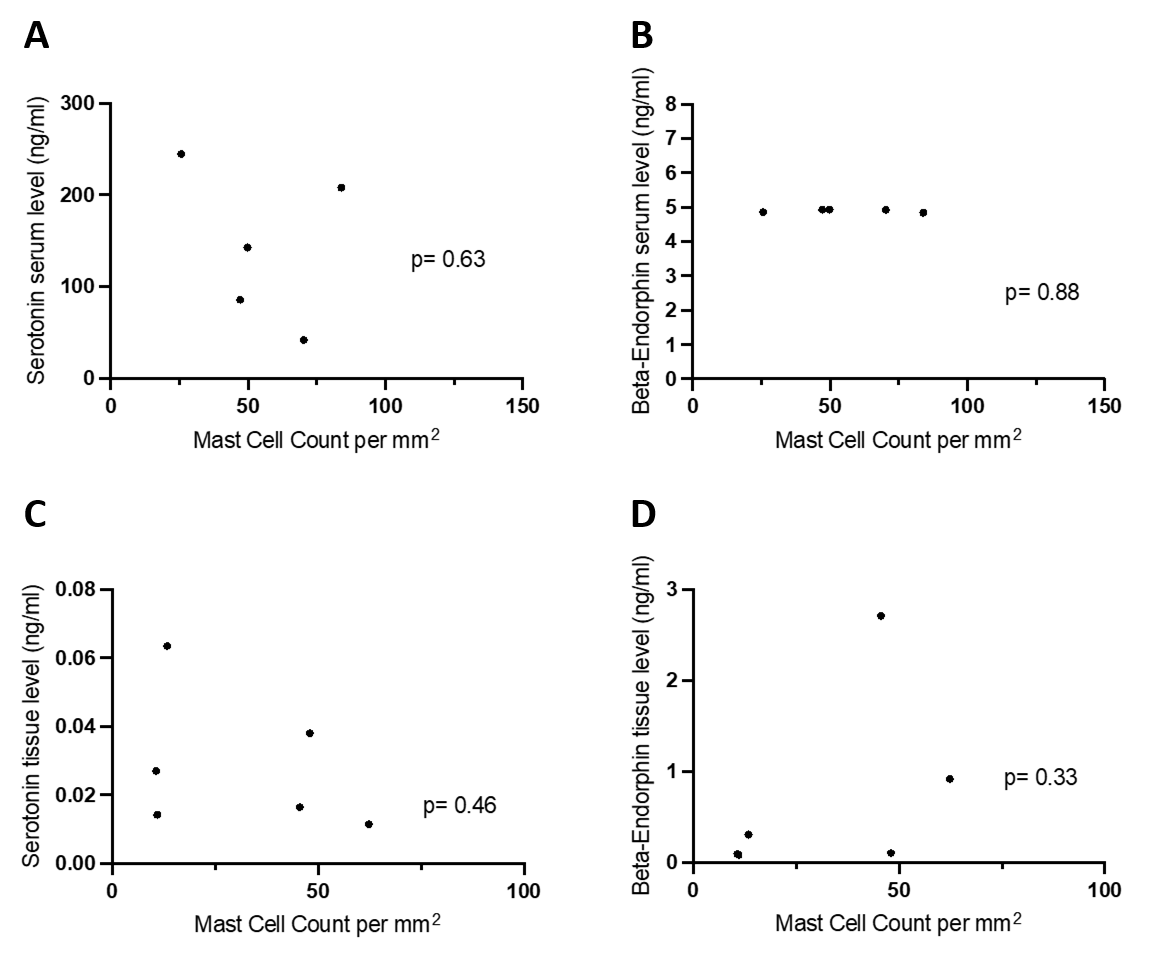


## Supplementary Table 1

| **Table S1: Correlation Of Patients Age With Distress, Depression And Anxiety Levels** | | | | | |
| --- | --- | --- | --- | --- | --- |
|  |  |  | **Distress** | **Depression level** | **Anxiety level** |
| **Spearman Rho** | **Age** | **ρ** | -0.168 | -0.255 | -0.326 |
|  |  | **p-value** | 0.314 | 0.265 | 0.149 |
|  |  | **n** | 38 | 21 | 21 |
| Depression and Anxiety levels were evaluated via HADS, Distress level via Distressbarometer | | | | | |

## Supplementary Table 2

| **Table S2: Comparison Of Qualitiy of Life Between Upfront Surgery and Neoadjuvant Chemotherapy Patients** | | | |
| --- | --- | --- | --- |
|  | **Upfront Surgery** | **Neoadjuvant Chemotherapy** | **p-value †** |
|  | n=29 | n=10 |  |
| **Physical functioning** |  |  |  |
|  | 69.5 ± 32.5 * | 74.2 ± 22.9 | 0.90 |
| **Physical role functioning** |  |  |  |
|  | 51.0 ± 44.7 ** | 32.5 ± 44.8 | 0.26 |
| **Pain** |  |  |  |
|  | 70.3 ± 29.8 | 76.3 ± 21.3 | 0.64 |
| **General health perception** |  |  |  |
|  | 53.7 ± 17.6 * | 50.5 ± 16.9 | 0.50 |
| **Emotional role functioning** |  |  |  |
|  | 58.3 ± 45.1 * | 53.3 ± 45.2 | 0.71 |
| **Vitality** |  |  |  |
|  | 50.2 ± 21.3 | 52.5 ± 17.2 | 0.74 |
| **Mental health** |  |  |  |
|  | 67.2 ± 18.9 | 62.8 ± 17.4 | 0.54 |
| **Social functioning** |  |  |  |
|  | 68.5 ± 33.4 | 68.8 ± 26.4 | 0.77 |
| **Comparison of health to the previous year** | |  |  |
|  | 25.0 ± 24.5* | 12.5 ± 20.2 | 0.15 |
| All data shown in mean ± SD, † Mann Whitney test, * n = 28 , ** n= 26 | | |  |

## Supplementary Table 3

| **Table S3: Correlation Of Mast Cells Count With Health-Related Quality Of Life** | | | |
| --- | --- | --- | --- |
|  | **n** | **Spearmann-Rho** | **p-value** |
| **Physical functioning** |  |  |  |
| Total Cohort | 38 | 0.07842 | 0.64 |
| Upfront Surgery | 28 | 0.09099 | 0.645 |
| Neoadjuvant Chemotherapy | 10 | - 0.01841 | 0.973 |
| **Physical role functioning** |  |  |  |
| Total Cohort | 36 | 0.1932 | 0.259 |
| Upfront Surgery | 26 | 0.2371 | 0.243 |
| Neoadjuvant Chemotherapy | 10 | - 0.104 | 0.779 |
| **Pain** |  |  |  |
| Total Cohort | 39 | - 0.02303 | 0.889 |
| Upfront Surgery | 29 | - 0.04074 | 0.834 |
| Neoadjuvant Chemotherapy | 10 | 0.01882 | 0.964 |
| **General health perception** |  |  |  |
| Total Cohort | 38 | 0.1396 | 0.403 |
| Upfront Surgery | 28 | 0.2431 | 0.212 |
| Neoadjuvant Chemotherapy | 10 | 0.006098 | 0.993 |
| **Emotional role functioning** |  |  |  |
| Total Cohort | 38 | 0.1347 | 0.42 |
| Upfront Surgery | 28 | 0.1092 | 0.58 |
| Neoadjuvant Chemotherapy | 10 | 0 |  |
| **Vitality** |  |  |  |
| Total Cohort | 39 | 0.2167 | 0.185 |
| Upfront Surgery | 29 | 0.2366 | 0.217 |
| Neoadjuvant Chemotherapy | 10 | 0.04878 | 0.897 |
| **Mental health** |  |  |  |
| Total Cohort | 39 | 0.2841 | 0.08 |
| Upfront Surgery | 29 | 0.2697 | 0.157 |
| Neoadjuvant Chemotherapy | 10 | 0.1885 | 0.6 |
| **Social functioning** |  |  |  |
| Total Cohort | 39 | 0.1744 | 0.288 |
| Upfront Surgery | 29 | 0.2478 | 0.195 |
| Neoadjuvant Chemotherapy | 10 | - 0.1595 | 0.658 |

## Supplementary Table 4

| **Table S4: Correlation of mast cell count with clinical characteristics** | | | | | | | | | | | | | |
| --- | --- | --- | --- | --- | --- | --- | --- | --- | --- | --- | --- | --- | --- |
|  |  |  | **BMI** | **diabetes mellitus** | **smoking** | **hypertension** | **jaundice** | **CA 19-9** | **leukocytes** | **hemoglobin** | **creatinine** | **albumin** | **bilirubin** |
| **Spearman Rho** | **Mast cell  count** | **ρ** | 0.020 | -0.071 | 0.006 | 0.004 | -0.009 | -0.166 | -0.009 | 0.098 | 0.159 | 0.027 | 0.022 |
|  |  | **p-value** | 0.902 | 0.664 | 0.973 | 0.979 | 0.957 | 0.379 | 0.957 | 0.549 | 0.327 | 0.891 | 0.893 |
|  |  | **n** | 40 | 40 | 40 | 40 | 40 | 30 | 40 | 40 | 40 | 28 | 40 |
| blood values taken on the day before surgery, BMI= body mass index, ρ = correlation coefficient | | | | | | | |  |  |  |  |  |  |
